# GanDouLing combined with Penicillamine improves cerebrovascular injury via PERK/eIF2α/CHOP endoplasmic reticulum stress pathway in the mouse model of Wilson’s disease

**DOI:** 10.1042/BSR20180800

**Published:** 2018-09-19

**Authors:** Yonghua Chen, Bo Zhang, Shijian Cao, Wei Huang, Ni Liu, Wenming Yang

**Affiliations:** 1 Department of Neurology, The First Affiliated Hospital of Anhui University of Chinese Medicine, Hefei, Anhui, China; 2 Graduate Institute, Anhui University of Chinese Medicine, Hefei, Anhui, China

**Keywords:** GanDouLing, Penicillamine, PERK/eIF2α/CHOP, WD

## Abstract

We aim to investigate the function and mechanism of GanDouLing combinated with Penicillamine on cerebrovascular injury in Wilson’s disease (WD). ELISA was performed to analyze the expression of vascular injury factors. Pathological changes of cerebral vessels were observed by HE stain. Immunohistochemistry assays were performed to analyze the expression of ICAM-1, VCAM-1, and GRP78. Western blotting was measured to analyze the expression of caspase-3, caspase-12, PERK, eIF2α, and CHOP. Apoptosis was detected with TUNEL assay. The expression of vascular injury factors and ICAM-1, VCAM-1 was significantly increased by WD and markedly decreased in GanDouLing-Penicillamine group. The expression of caspase-3, caspase-12, PERK, eIF2α, and CHOP were obviously expressed in Wilson group, GanDouLing-Penicillamine suppressed apoptosis and endoplasmic reticulum (ER) stress. Our findings suggested that GanDouLing-Penicillamine improved cerebrovascular injury through PERK/eIF2α/CHOP ER stress pathway in the mouse model of WD.

## Introduction

Wilson’s disease (WD) was first described by Kinnear Wilson in 1912 [1], which is an autosomal recessive metabolic disease caused by a mutation of *ATP7B* gene [2,3]. The *ATP7B* gene is a copper-transporting P-type ATPase (WD protein), mutation of this gene leads to the accumulation of copper in various tissues and organs, especially liver, brain, cornea, and kidneys [4]. Clinical manifestations vary from an asymptomatic state to acute hepatic failure, chronic liver disease, and neurological symptoms such as dystonia, tremor, dysarthria, and psychiatric disturbances [5,6]. WD has high lethality, but it is one of the few diseases that can be treated in congenital genetic diseases. Early diagnosis and timely treatment are conducive to the effective control, whereas poor prognosis even life-threatening [7]. Therefore, it is meaningful and valuable to investigate the drug treatment of WD and the mechanism of related treatment in the clinic.

Endoplasmic reticulum (ER) is a crucial site for protein modification, folding, and calcium storage. ER stress is considered as an imbalance between the protein folding capacity of the organelle and the functional demand due to different physiological and pathological perturbations interfere, such an imbalance leads to accumulation of unfolded or misfolded proteins in the ER lumen [8]. To revivify ER homeostasis, the ER responds to stress by activating intracellular signal transduction pathways, collectively intituled as the unfolded protein response (UPR) [9,10]. The UPR might alleviate the ER stress through regulating different transcription pathways [11–14]. It has been pointed out that ER stress is involved in the pathophysiological processes of multiple vascular injury diseases such as atherosclerosis, hypertension, vascular calcification, and restenosis after coronary angioplasty [15,16]. To date, there have been no reports on whether WD has an effect on cerebral vessels or the disease is related to ERs.

The treatment of WD is based on the removal of copper excess by chelating agents such as trientine, tetrathiomolybdate or by blocking the intestinal copper absorption with zinc salts; however, their elevated cost or adverse effects might hamper use in clinic [17]. At present, Penicillamine (d-Penicillamine), is widely used due to its low cost and considerable efficacy [18,19]. Accumulating studies have indicated the therapeutic effect of *Penicillium* in liver disease [20,21], especially WD [19,21]. However, there are also many adverse reactions, such as fever, rashes, gastrointestinal reaction, leukocyte reduction, systemic lupus erythematosus, nephritis etc, which limits its clinical application [6]. Amount of clinical practice and theoretical studies have proved that traditional Chinese medicine (TCM) is medicative for WD. TCM can eliminate abnormal deposition of copper ions in liver, brain, and other tissues not only by renal metabolic pathways but also bile secretion, which improves curative effect and reduces the side effects of Western medicine; more importantly, the combined application of Chinese and Western medicine is better than that of Western medicine alone [22]. GanDouLing is a Chinese medicinal herb produced by the preparation center of Anhui Hospital of TCM, which is mainly composed of turmeric, radix curcumae, rhizoma coptidis, rhizoma zedoariae, *Salvia miltiorrhiza*, caulis spatholobi, and *Rheum officinale*. GanDouLing and its composition play important roles in removing blood stasis, invigorating blood circulation, and promoting copper excretion in liver and gallbladder. For instance, multiple studies have validated turmeric’s list of medicinal uses, which include being an antioxidant [23], anticarcinogenic [24], and hepatoprotective [25]. Radix curcumae (Yujin) with obvious pharmacological activity, has been used for approximately 1300 years in China, is a kind of Chinese medicinal herbs for treating hepatic diseases [26]. Our previous studies have shown that GanDouLing has a good effect on improving the neurological function of WD patients; here, we further proved the combined application of GanDouLing and Penicillamine has more beneficial effects, whereas specific mechanism of GanDouLing combinated with Penicillamine needs further exploration.

## Materials and methods

### Ethics statements

Forty-eight females at the age of 8–10 weeks, toxic milk (TX) mice (20 ± 2 g) were obtained from the Experimental Animal Centre of Anhui. The present study was carried out in strict accordance with the recommendations of the Guide for the Care and Use of Laboratory Animals of the National Institutes of Health. The animal use protocol was reviewed and approved by the Institutional Animal Care and Use Committee of Anhui hospital of TCM.

### Establishment of animal models

Forty-eight females TX mice were divided into four groups, 12 mice in each group: Wilson group, GanDouLing group (0.486 g.kg^−1^.day^−1^, GanDouLing was produced by Anhui Hospital of TCM: the active ingredients of each herb were extracted with 65% ethanol and then combined with extracted filtrate. The filtrate combinations were baked into dry paste at right temperature, starch was added and packed into GanDouLing troche). Penicillamine group, (0.09 g.kg^−1^.day^−1^, Anhui Hospital of TCM) and GanDouLing (0.243 g.kg^−1^.day^−1^) -Penicillamine (0.045 g.kg^−1^.day^−1^) group. Twelve female DL mice (20 ± 2 g) from The Animal Experimental Center in Anhui were treated as control group. The mice in GanDouLing groups were treated by intragastric administration with GanDouLing in 0.486 g/kg every day for 8 weeks. The mice in Penicillamine groups were treated by intragastric administration with Penicillamine in 0.09 g/kg mixed with distilled water every day for 8 weeks. Wilson group and control group were treated by intragastric administration with an equivalent volume of 0.9% saline every day. All the mice were housed in a controlled humidity (50–70%) and room temperature (18–22°C), fed in the isolation cages with independent air supply, and were given free access to food and water *ad libitum* in an alternating 12-h light/dark cycle over a period of 8 weeks.

### ELISA

The blood sample was collected from the abdominal aorta. The supernatant was measured with von Willebrand factor (vWF), TM, and ACA ELISA kits (Nanjing Jiancheng Bioengineering Institute, Nanjing, China) according to instructions. The concentration was calculated according to the corresponding OD value.

### Hematoxylin and Eosin stain

The rat brain isolated tissues were fixed with 4% paraformaldehyde for 3 h and then dehydrated with ethanol and xylene. The brain samples were embedded in paraffin and sliced into 4-μm sections, tissue sections were deparaffinized in xylene and hydrated through graded ethanols. After that, sections were dipped into hematoxylin buffer and eosin buffer (Solarbio, Beijing, China), and then washed with water. The sections were dehydrated in a gradient ethanol series and xylene, and were then covered into watch and analyzed under light microscopy (Metamorphy/BX51, UIC/Olympus, U.S.A./JAP). The result of staining was that the cell nucleus was dyed blue by Hematoxylin, and the cytoplasm was dyed red by Eosin.

### Immunohistochemistry assay

The 4-μm sample sections were deparaffinized in xylene and hydrated through graded ethanols. The sections were incubated with H_2_O_2_ deionized water and rinsed with distilled water. Then, antigen retrieval was carried out with citrate buffer by heating for 15 min in a microwave. The primary antibodiesm, including ICAM-1 (ICAM-1 polyclonal anti-rabbit antibodies, 1:100, Abbiotec LCC San Diego, CA, U.S.A.), VCAM-1 (VCAM1 rabbit polyclonal antibody, 1:100 Abbiotec LCC San Diego, CA, U.S.A.), and GRP78 (1:100, Proteintech, U.S.A.) were added and incubated overnight at 4°C and rinsed with PBS, and then the second antibodies were added and incubated at 37°C for 2 h and rinsed with PBS. The sections were dipped into chromogenic agent and washed with water. They were dehydrated in a gradient ethanol series and xylene, and then covered. The immunohistochemical results were observed.

### TUNEL staining

TUNEL staining was performed for sample paraffin sections using an *in situ* cell death detection kit (Intergen Co., U.S.A.) according to the manufacturer’s instructions. After dewaxing and aquation, slices were permeabilized in proteinase K (20 μg/ml) for 20 min at room temperature. The slices were then exposed to TdT equilibration buffer, recombinant TdT enzyme, and Alexa Fluor 488-12-dUTP labeling mix for 60 min at 37°C. Nuclear staining was identified in cell nuclei with DAPI.

### Western blot

The brain lining tissue samples were homogenated in RIPA buffer with PMSF on ice. Total protein was extracted and the concentration was detected by BCA Kit (Beyotime Biotechnology). Polyacrylamide gel is prepared according to the specification of SDS/PAGE Gel Preparation Kit (Beyotime Biotechnology). Electrophoresis was carried out in the SDS/polyacrylamide gel using 20 μg of total protein in each lane (Tanon, Shanghai, China). After electrophoresis, protein was transferred to a PVDF membrane. Then the membrane was incubated with corresponding primary antibodies including caspase-3 (1:500, Genetex, U.S.A.), caspase-12 (1:500, Genetex, U.S.A.), PERK (1:500, Bioworld Technology, U.S.A.), CHOP (1:500, Bioworld Technology, U.S.A.), eIF2α (1:1000, Abcam, U.K.), and phosphorylated eIF2α (p-eIF2α) (1:1000, Proteintech, U.S.A.) overnight at 4°C. After washing with PBS–0.1% Tween, the membrane is incubated with secondary antibodies conjugated with horseradish peroxidase for 2 h at room temperature. The membrane is washed again and protein is detected by ECL (Thermo Scientific SuperSignal West Pico). The relevant factors were represented by the relative yield to the GAPDH (1:1000, ZSGB-BIO, China).

### Data analysis

All the statistical analyses were analyzed with SPSS 22.0 software, the correlations between the parameters of samples were assessed by Pearson’s test. Student’s *t* test was used to analyze differences between two groups. One-way ANOVA analysis was used to determine the multisample analysis. All statistical tests were two-sided, and statistical significance was defined as *P*<0.05.

## Results

### WD induced cerebrovascular injury in the mouse model

**Figure 1:**
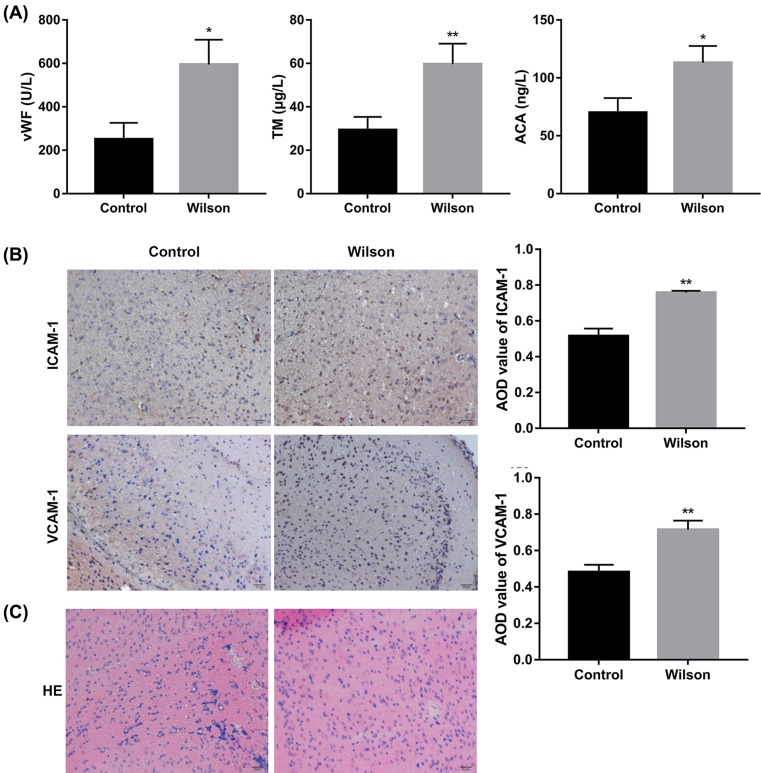
WD induced cerebrovascular injury (**A**) ELISA results of vascular injury factors (vWF, TM, ACA) in serum in control and Wilson groups. (**B**) Immunohistochemistry of ICAM-1 and VCAM-1 of cerebrovascular endothelial cells in control and Wilson groups. (**C**) The cerebrovascular injury induced by WD by HE in control and Wilson groups (*P<0.05, **P<0.01, compared with control).

vWF was considered as a marker of endothelial cell injury to evaluate the function and injury of vascular endothelial cells [27]. Thrombomodulin (TM) is a marker of vascular endothelial injury and destruction [28]. Anti-cardiolipin antibody (ACA) can directly cause the injury of vascular endothelial cells via stimulating organisms to produce an autoimmune antibody with strong coagulation activity [29]. ICAM-1 and VCAM-1 induced endothelial cell injury in the inflammatory response of vascular injury [30,31]; therefore, ICAM-1 and VCAM-1 were considered as markers for vascular injury in this experiment. As seen in Figure 1A, the expression of vWF, TM, and ACA was markedly higher in Wilson group than that in control group. Immunohistochemical results showed that there was an obvious positive expression of ICAM-1 in Wilson group, but not in control group, and the positive reactions were mainly tan or brown on the vascular. Similarly, VCAM-1 positive staining was not observed in the control group, but there was significant expression of VCAM-1 in Wilson group, which appeared tan on the vascular wall (Figure 1B). HE staining was observed with the light microscope, and the cerebral vascular endothelial cells in Wilson group appeared edema degeneration, but not so in the control group (Figure 1C). The results indicated that WD induced cerebrovascular injury in the mouse model.

### Action observation of GanDouLing combinated with Penicillamine on cerebrovascular injury in the mouse model

**Figure 2:**
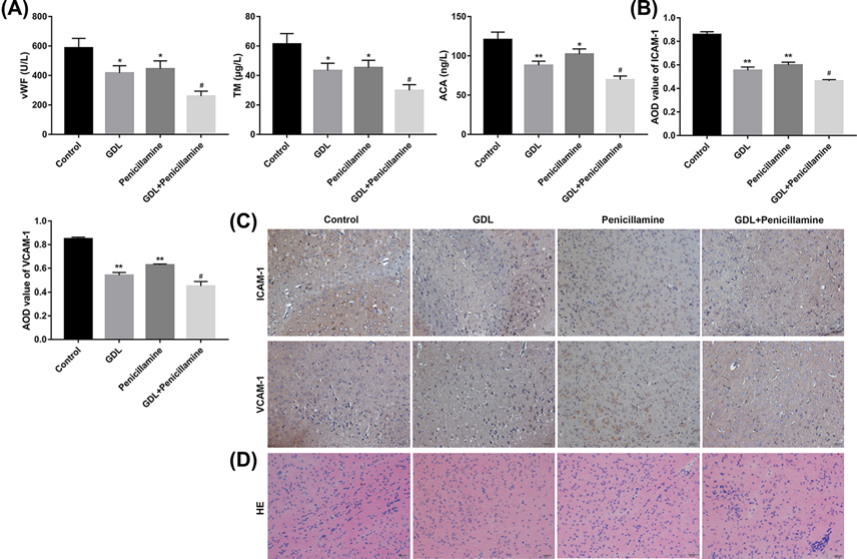
Action observation of GanDouLing combinated with Penicillamine on cerebrovascular injury (**A**) ELISA results of vascular injury factors (vWF, TM, ACA) in serum in control, GDL, Penicillamine, and GDL+Penicillamine group. (**B**,**C**) Immunohistochemistry of ICAM-1 and VCAM-1 of cerebrovascular endothelial cells. (**D**) The cerebrovascular injury induced by WD by HE in serum in control, GDL, Penicillamine, and GDL+Penicillamine group (*P<0.05, **P<0.01, compared with control; ^#^*P*<0.05, compared with Penicillamine).

Compared with control group, the expression of vWF, TM, and ACA was markedly decreased in GDL group, Penicillamine group, and GDL-Penicillamine groups, and the expression of vascular injury factors was decreased in the combination group (Figure 2A). The results of immunohistochemistry in cerebrovascular endothelial cells showed that compared with Penicillamine or GDL group, the expression of ICAM-1 and VCAM-1 significantly reduced in the GDL-Penicillamine group (Figure 2B,C). The pathological morphology changes showed more slight edema degeneration occurred in GDL group and Penicillamine group in comparison with control group (Figure 2D). Collectively, the results indicated that GanDouLing and Penicillamine improve cerebral vascular injury, the therapeutic effect of GanDouLing was superior to *Penicillium*, and the combination therapy of GanDouLing and Penicillamine worked best in WD mice.

### The effect of GanDouLing combinated with Penicillamine on apoptosis of cerebrovascular tissue and ERS

**Figure 3:**
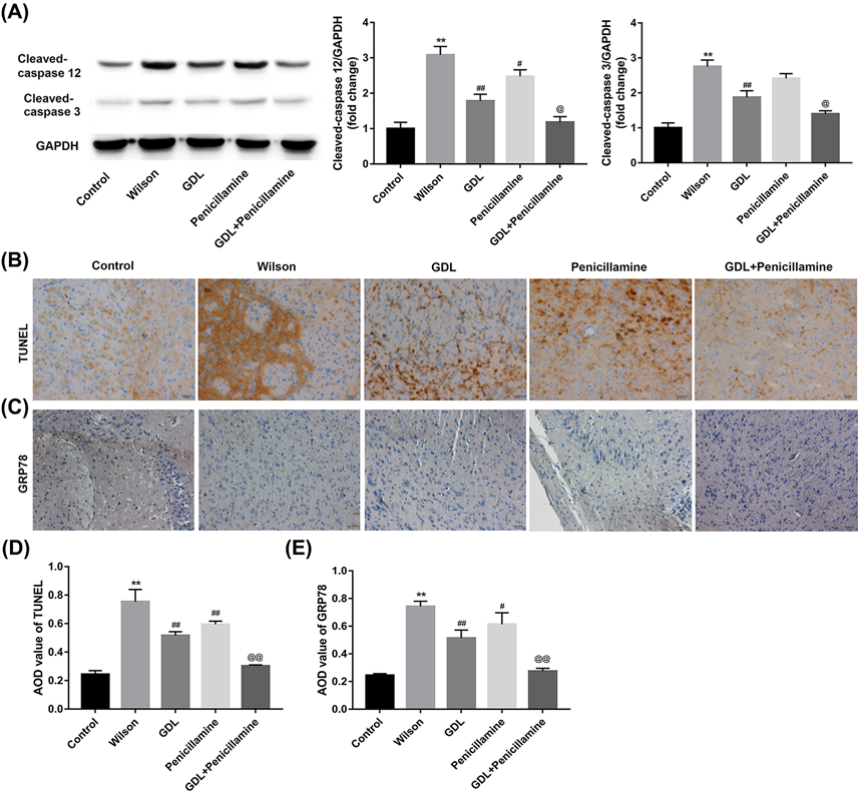
The effect of GanDouLing combinated with Penicillamine on apoptosis in the cerebrovascular tissue and ERS (**A**) Western blotting result of caspase-3, caspase-12 in the brain lining tissue. (**B**,**D**) TUNEL result of apoptosis in cerebrovascular endothelial cells. (**C**,**E**) Immunohistochemistry of GRP78 of cerebrovascular tissue (**P<0.01, compared with control; ^#^*P*<0.05, ^##^*P*<0.01 compared with Wilson; ^@^*P*<0.05, ^@@^*P*<0.01 compared with Penicillamine).

To investigate the apoptosis in cerebrovascular injury, we detected the apoptotic factors caspase-3 and caspase-12. In comparison with control group, caspase-3 and caspase-12 are obviously expressed in Wilson group, GanDouLing and Penicillamine suppressed the expression of caspase-3 and caspase-12, and the combination of them achieved maximal inhibition effect (Figure 3A). Compared with control group, the tendency of the TUNEL changes on apoptosis is similar with the above findings (Figure 3B,D). The above results indicated that GanDouLing and Penicillamine can inhibit apoptosis of cerebrovascular endothelial cells, and the inhibition effect of GanDouLing-Penicillamine was stronger than that of single drug. GRP78 is one of the ERS signals. In our findings, there was obvious ERS in Wilson group, GanDouLing and Penicillamine played significant roles in inhibiting ERS; similarly, the combination of the two worked most markedly (Figure 3C,E).

### Intervention pathway of GanDouLing-Penicillamine on ERS

**Figure 4:**
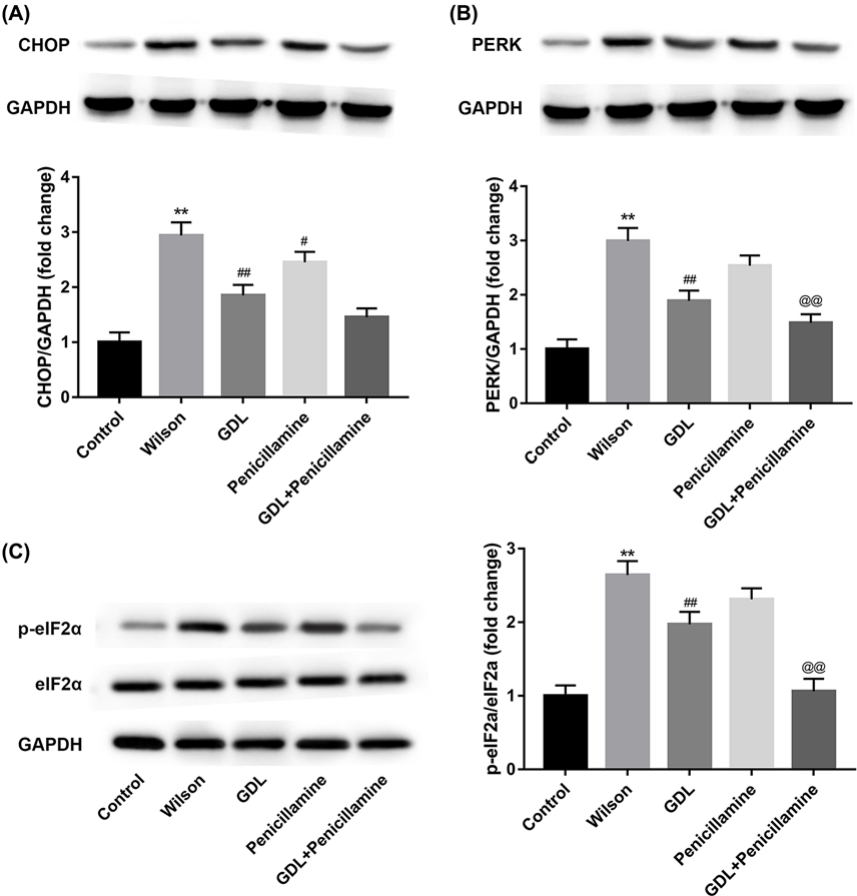
Intervention pathway of GanDouLing combinated with Penicillamine on ERS (**A**) Western blotting result of CHOP in brain arterial intima. (**B**) Western blotting result of PERK. (**C**) Western blotting result of eIF2α and p-eIF2αin brain arterial intima (**P<0.01, compared with control; ^#^*P*<0.05, ^##^*P*<0.01 compared with Wilson; ^@@^*P*<0.01 compared with Penicillamine).

PERK, an ER stress sensor protein, can directly interact with misfolded proteins to induce ER stress signaling [32]. ERS can enhance apoptosis via CHOP pathway [33]. To further investigate the intervention pathway of GanDouLing-Penicillamine on ERS, the protein levels of CHOP, PERK, and eIF2α were detected by Western blotting. The expression of CHOP and PERK is significantly increased by Wilson, GanDouLing, and Penicillamine, as well as GanDouLing-Penicillamine markedly suppressed the expression of CHOP and PERK, respectively, (Figure 4A,B). The phosphorylation level of eIF2α is similar with the above findings of CHOP and PERK (Figure 4C). In conclusion, the results demonstrated that GanDouLing combinated with Penicillamine-inhibited ERS through PERK/eIF2α/CHOP pathway.

## Discussion

In the present study, we identified cerebral vascular injury of mice caused by WD at the first attempt. Furthermore, we found that GanDouLing could improve cerebrovascular injury of mice, and it worked better on combined application with *Penicillium*. Compared with the control group, the expression of GRP78 and PERK signaling protein (PERK, eIF2α, and CHOP) in the GanDouLing-*Penicillium* group was markedly up-regulated, and apoptosis indicators (caspase-3 and caspase-12) were obviously increased, TUNEL assay showed significant increase in apoptosis of cerebrovascular endothelial cells, which suggested that GanDouLing-*Penicillium* improves cerebrovascular injury through PERK/eIF2α/CHOP ER stress pathway in the WD mouse model.

Hawkins et al. [34] found that there was low blood flow perfusion in the cerebral cortex of WD patients through PET examination, Smith et al. [35] reported that regional cerebral blood flow of the bilateral thalamus decreased in WD patients. The above reports indicated that there was cerebrovascular injury in WD patients, which is consistent with our research results in WD mice. In addition, Penicillamine was effective for treating WD mice [36,37], and we further found that the efficacy of combined treatment of GanDouLing and Penicillamine is superior to their monotherapy in WD mice.

The ER is responsible for protein synthesis and folding [38]. Different physiological and pathological interfere with protein-folding processes in the ER lumen can affect the function of ER, leading to ERS [39]. The ER responds to stress by activating intracellular signal transduction pathways [39]. GRP78, an ER chaperone protein, regulates protein translocation, folding, and degradation of the misfolded protein [40] to restore the homeostasis of ER. Caspase-3 is an executioner in caspase cascades, which are of much concern on apoptosis-induced cell death [41]. Caspase-12 is the initial factor of dependent cell apoptosis pathway. The present study results of significantly overexpressed caspase-3, caspase-12, GRP78, and TUNEL analysis of apoptosis in Wilson group demonstrated that the caspase pathway was activated by lasting ERS of WD-induced cerebrovascular injury. Compared with the Penicillamine group, GRP78 expression was significantly reduced in GanDouLing-Penicillamine group, which indicated that the combination therapy of GanDouLing and Penicillamine maximally alleviated cell apoptosis and improved cerebral vascular injury by inhibiting ER stress.

The ES response included the adaptive response phase and the apoptosis stage [42]. PERK is the main signal of ERS and UPR. The protein eIF2α, a downstream target of PERK, is regulated by the occurrence of ERS [43,44]. EIF2α will inhibit protein synthesis and protect cellular sources while stress-induced damage is prevented by gene expression [45]. The p-eIF2α reduces the influx of nascent proteins into the ER lumen and enhances the capacity for degradation of misfolded protein [45]. When the cell is underwent by severe or lasting stimulus, ERS is unable to restore function through above adaptive response, thereby induced apoptosis [46]. CHOP is one of the pathways to induce apoptosis, the activation of CHOP is regulated by PERK/eIF2a, and p-eIF2a activates the downstream transcription factor ATF4, which further up-regulates the expression of downstream gene CHOP [42]. Curcumin [1,7-bis-(4-hydroxy-3-methoxyphenyl)-1,6-heptadiene-3,5-dione] is a major component of turmeric, which has been used to treat a broad range of ailments. Curcumin has an inhibitory effect on ER stress in its role as an antioxidant [47]. Previous studies have demonstrated turmeric regulate ER stress signaling through GRP78, CHOP, p-eIF2α, and p-PERK in CCl_4_-induced hepatic toxicity [48]. Therefore, we hypothesized that turmeric, as major component of GanDouLing, is responsible for the inhibition of ER stress.

PERK/eIF2α/CHOP is an essential signaling pathway that regulates ER stress [49]. In our study, the expression of CHOP, PERK, and eIF2α were significantly increased by Wilson, GanDouLing and Penicillamine effectively down-regulated proteins expression, and proteins expression most obviously reduced in the combination group. Given that, the results demonstrated that GanDouLing combinated with Penicillamine inhibited ERS through PERK/eIF2α/CHOP pathway.

## Abbreviations

ACA: anti-cardiolipin antibody
ATF4: activating transcription factor 4
ER: endoplasmic reticulum
eIF2α: the α subunit of eukaryotic translation initiation factor 2
GDL: GanDouLing
HE: Hatmatoxylin-Eosin
ICAM-1: intercellular adhesion molecule-1
p-eIF2α: phosphorylated eIF2α
PERK: protein kinase RNA-like endoplasmic reticulum kinase
RIPA: radio immunoprecipitation assay
TCM: traditional Chinese medicine
TM: thrombomodulin
TX: toxic milk
UPR: unfolded protein response
VCAM-1: vascular cell adhesion molecule-1
vWF: von Willebrand factor
WD: Wilson’s disease
